# Predictive Equation for Angle Opening Distance at 750 μm After Laser Peripheral Iridotomy in Primary Angle Closure Suspects

**DOI:** 10.3389/fmed.2021.715747

**Published:** 2021-08-12

**Authors:** Xinbo Gao, Yuying Zhou, Chengguo Zuo, Liming Chen, Jiawei Ren, Huishan Lin, Yunru Liao, Haijun Gong, Huanling Hu, Mingkai Lin

**Affiliations:** State Key Laboratory of Ophthalmology, Zhongshan Ophthalmic Center, Sun Yat-sen University, Guangzhou, China

**Keywords:** LPI, UBM and glaucoma, angle opening distance, primary angle closure suspects, predictive equation

## Abstract

**Aim:** The aim of this study was to investigate the changes in anterior segment parameters as assessed by ultrasound biomicroscopy (UBM) after laser peripheral iridotomy (LPI) and to propose a prediction equation for the width of the angle after LPI.

**Design:** This was a prospective study.

**Participants:** The participants included 100 subjects with primary angle closure suspect (PACS).

**Methods:** Anterior segment UBM parameters were measured, whereas AOD750 was chosen to indicate the width of the angle associated with gonioscopic angle closure, as found in a prior study.

**Main Outcome Measures:** Angle parameters, iris parameters, anterior chamber parameters and ciliary body parameters.

**Results:** All angle parameters increased after LPI, including the mean angle opening distance at 750 μm (AOD750), mean angle opening distance at 500 μm from the scleral spur (AOD500), mean angle opening distance at 750 μm from the scleral spur (AOD750), and mean angle recess area at 750 μm from the scleral spur (ARA750). Among iris parameters and ciliary body parameters, the iris thickness at 2,000 μm (IT2000), iris curvature (IC), and trabecular-ciliary process distance (ICPD) were reduced after LPI. The final equation consisted of four parameters: anterior chamber depth (ACD), iris thickness at 750 μm from the scleral spur (IT750), AOD750, and lens vault (LV). This equation explained 42.7% of the variability in the angle opening indicator AOD750 after LPI, whereas in the plateau iris configuration subgroup, the accuracy of the prediction equation reached the highest a maximum of 68.6%.

**Conclusions:** There was an increase in angle opening and iris flattening after LPI. An equation involving four angle parameters was constructed, this equation which could explained 42.7% of the variability in the angle opening indicator AOD750 after LPI whereas in the plateau iris configuration subgroup, the accuracy of the prediction equation reached a maximum of 68.6%.

## Introduction

The incidence of angle-closure glaucoma is between 1.1% and 1.6% in China. By 2050, the total incidence of glaucoma in China may reach 3.48% (1). The prevalence of primary angle-closure suspect (PACS) is significantly higher than that of primary angle-closure (PAC) or primary angle-closure glaucoma (PACG) (2). Laser peripheral iridotomy (LPI) is an option for the treatment of PACS because it can establish a channel, relieve the pupil block component, and flatten the iris plane, thus increasing the width of the anterior chamber angle (3, 4). However, the effect of LPI on the induction of angle opening is not consistent, and the actual role of LPI is still controversial. Twenty percent of PACS patients still had iridotrabecular contact after LPI treatment (3). A previous study found that for every 0.1 mm decrease in the angle opening distance at 750 μm from the scleral spur (AOD750), the odds of developing gonioscopic angle closure increased by a factor of 3.27 (5). To predict the post-procedural effect of LPI, it is necessary to establish a prediction equation for the degree of angle opening after LPI based on the morphological structure of the eye.

As an objective and relatively reliable imaging method, ultrasound bomicroscopy (UBM) can reproducibly measure the parameters of the anterior chamber. Recent studies have explored the correlation between LPI-induced angle widening and baseline parameters (including angle parameters, anterior chamber parameters, iris parameters, and others) (6). The predictors of angle opening after LPI were identified as baseline central anterior chamber depth (cACD), AOD750, iris curvature (IC) and lens vault (LV) (7–9), and their proposed prediction accuracy for post-procedural angle opening distance based on pre-procedural baseline parameters ranged from 10 to 40%. However, the equation to predict the angle opening distance after LPI did not have high predictive value. In this study, we used UBM to establish the relationship between the angle opening distance after LPI and baseline parameters, and we proposed a predictive equation for the angle opening distance after LPI to predict the post-procedural effect of this technique.

## Materials and Methods

### Design and Participants

All tests and treatments were carried out at the Zhongshan Ophthalmic Clinical Research Center, a tertiary hospital in Guangzhou, China. The experiment was approved by the Ethical Committee of Zhongshan Ophthalmic Center. The experiment was carried out according to the principles of the Declaration of Helsinki. From September 2019 to September 2020, patients diagnosed with PACS who underwent LPI were enrolled. All the participants provided written informed consent. The inclusion criteria were as follows: (1) PACS was diagnosed according to International Society for Geographical and Epidemiological Ophthalmology (ISGEO) definition, in which patients with narrow angles that ≥6h clock hours circumstances pigmented trabecular meshwork was not visible under non-indentation gonioscopy); (2) no peripheral anterior iris synechiae were found on gonioscopic examination; (3) intraocular pressure (IOP) ≤ 21 mmHg; and (4) no abnormalities were found on fundus examination. The exclusion criteria included serious general health problems, previous intraocular surgery, previous iridoplasty, and inability to undergo LPI due to the opacity of the media.

### Pre-procedural and Post-procedural Evaluation

All patients underwent routine ophthalmic examinations, including the Humphrey visual field examination, IOP examination, slit-lamp examination, gonioscopy, and UBM examination. An experienced ophthalmologist performed gonioscopy in a dark room with a slit lamp. The Scheie method was used to classify the angle.

Two senior glaucoma experts, associate chief physician Zuo Chengguo and attending physician Gao Xinbo, divided PACS patients into pupillary block type, a plateau iris configuration, and a mixed mechanism type according to their UBM images before LPI. If the two glaucoma doctors disagreed on a patient's classification, a third senior glaucoma expert, chief physician Lin Mingkai, made the final judgement. We distinguished the mechanism of angle closure according to the following definitions (10):

1. Pupillary block angle type: iris bombe alone is present in at least two quadrants.
2. Plateau iris configuration: the iris profile is flat, and the iris root is shifted forward as a result of anterior positioning of the ciliary processes, making the iridocorneal angle narrow and the anterior chamber deep.
3. Mixed mechanism angle type: a combination of the pupillary block angle type and the plateau iris configuration.

**Figure 1:**
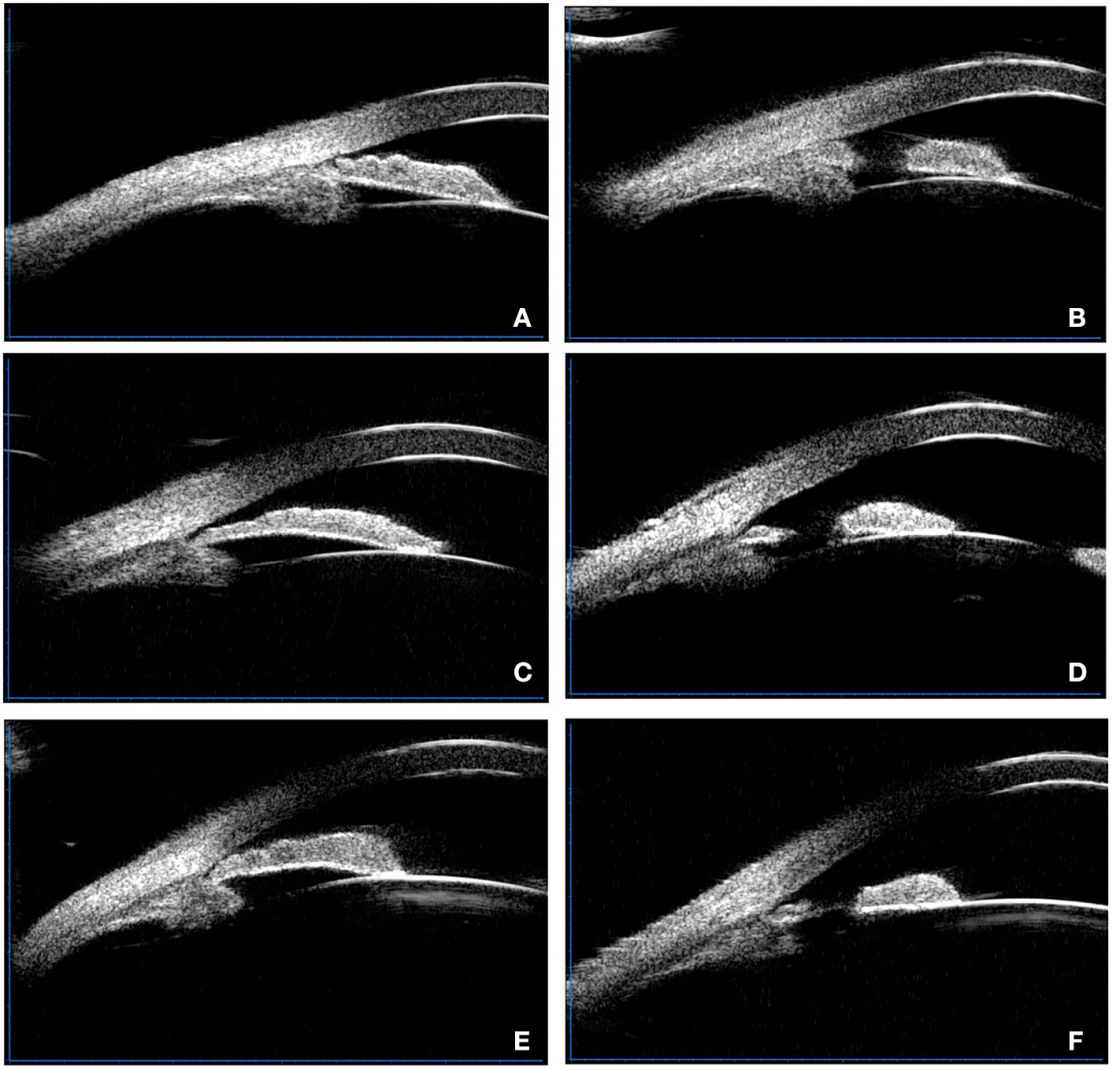
Illustrated the three types of angle-closure mechanisms before and after LPI. **(A)** Typical plateau iris configuration before LPI, the peripheral iris is thick. **(B)** Typical plateau iris configuration after LPI, the peripheral iris is still thick. **(C)** Typical pupillary block type picture before LPI, iris bombe can be observed. **(D)** Typical pupillary block type picture after LPI, the iris is flattened. **(E)** Typical mixed mechanism type before LPI. **(F)** Typical mixed mechanism type after LPI, the iris is flattened slightly.

One of the glaucoma specialists treated this cohort using a VISULAS® 532s diode laser (Carl Zeiss Meditec, Inc.) Two percent pilocarpine eye drops were used for pretreatment. LPI was performed at the thinnest part of the iris (at the 10–12 o'clock and 12–2 o'clock positions). The signs of full-thickness perforation were the forward movement of aqueous matter from the posterior chamber to the anterior chamber and the dispersion of pigment. The diameter of the perforation was approximately 200 μm. Immediately after surgery, all eyes were immediately treated with prednisolone and at least one drug to lower intraocular pressure. All patients were given 1% prednisolone six times on the day of LPI and six times a day for the next week. Two to three weeks after LPI, we obtained and evaluated UBM images. Figure 1 illustrated the three types of angle-closure mechanisms before and after LPI.

### Ultrasound Biomicroscopy Examination

A technician performed ultrasound biomicroscopy (SW-3200L, Suoer, Tianjin) before and after LPI. When the subjects gazed at the calibration object with the opposite eye, we obtained four-quadrant angle structure images and one central anterior chamber image. From 12 o'clock, the eyeballs of all patients were examined clockwise (the visual angle was kept at approximately 20 degrees, and the control was adjusted). After topical anesthesia, the probe was placed perpendicular to the ocular surface, and normal saline was used as the coupling agent. The gain was set between 60 and 80 dB to maximize the field of view of the imaging structure and minimize “noise”. We attempted to ensure that the scleral spur, the angle, the ciliary body, and half the iris chord length were clearly visible.

### Qualitative and Quantitative Analysis of Ultrasound Biomicroscopy

All UBM parameters namely, anterior chamber depth (ACD), anterior chamber width (ACW), LV, IC, trabecular-ciliary process distance at 750 μm (TCPD750), iris-ciliary process distance at 750 μm (ICPD750), ciliary body thickness (CBT0), angle recess area (ARA), iris thickness at 750 μm (IT750), and iris thickness (IT2000), were obtained using the UBM technique (SW-3200L, Suoer, Tianjin).

**Figure 2:**
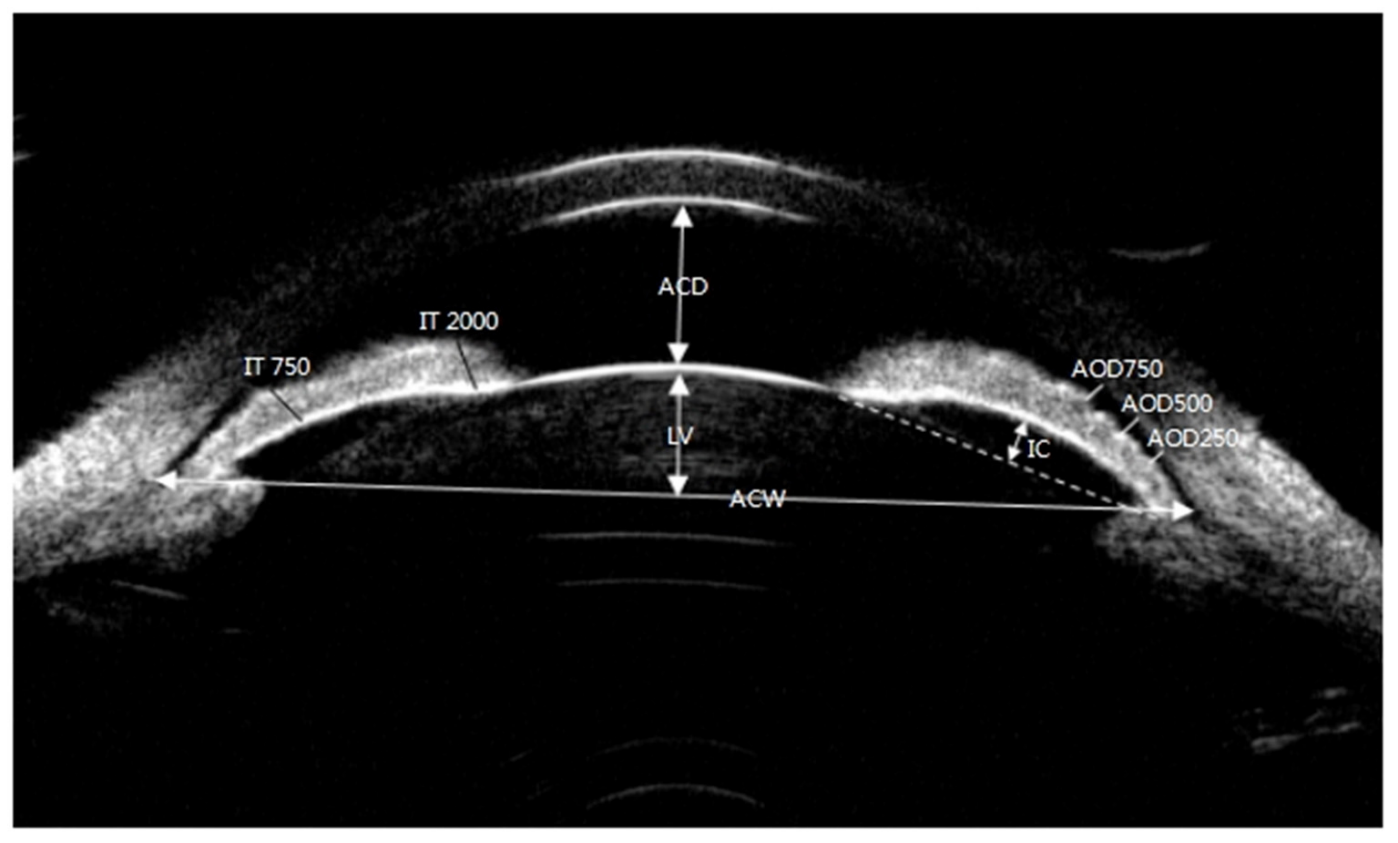
Ultrasound biomicroscopic (UBM) images of ACD, LV, ACW, AOD250, AOD500, AOD750, IT750, IT2000, and IC. ACD, anterior chamber depth; LV, lens vault; ACW, anterior chamber width; AOD250, angle opening distance at 250 μm from the scleral spur; AOD500, angle opening distance at 500 μm from the scleral spur; AOD750, angle opening distance at 750 μm from the scleral spur.

**Figure 3:**
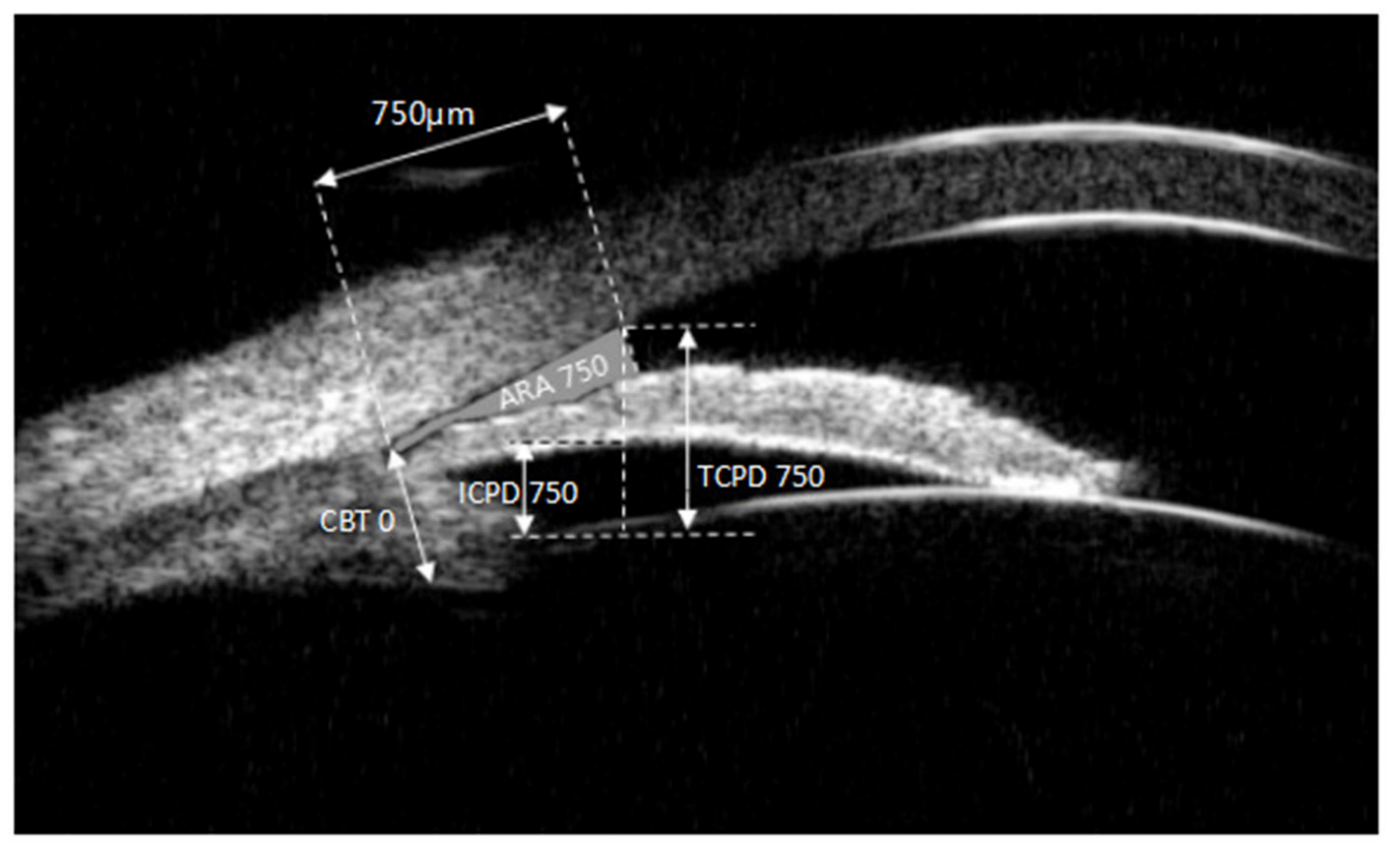
Ultrasound biomicroscopic (UBM) images ARA750, ICPD750,TCPD750, CBT0. ARA750, angle recess area at 750 μm from the scleral spur; ICPD750, iris-ciliary process distance at 750 μm; TCPD750, trabecular-ciliary process distance at 750 μm; CBT0, ciliary body thickness at the point of the scleral spur.

Figures 2, 3 illustrated their calculation method, the ACD was defined as the axial distance between the corneal endothelium and the anterior lens surface. The ACW was defined as the distance between the two scleral spurs (SS). The LV was defined as the perpendicular distance from the anterior pole of the lens to the horizontal line between the scleral spurs. The IC was defined as the vertical distance between the highest point of the back bulge of the iris and the line between the iris root and the edge of the pupil. TCPD750 was defined as a line extending from the corneal endothelium 750 μm anterior to the SS toward the ciliary processes. ICPD750 was defined as the posterior surface of the iris 750 μm anterior to the SS toward the ciliary processes. CBT0 was defined as ciliary body thickness at the point of the SS. Anterior opening distances of 250, 500, and 750 μm were measured on a line perpendicular to the plane of the trabecular surface 250, 500, and 750 μm, anterior to the scleral spur and extended to meet the surface of the iris. The ARA was the area bounded by the anterior surface of the iris surface, corneal endothelium, and the line perpendicular to the trabecular surface plane to the iris surface, starting from 750 μm before the scleral puncture. IT750 and IT2000 are defined as iris thickness at 750 and 2,000 μm, respectively, from the SS.

### Statistical Analysis

SPSS software (IBM SPSS Statistics 23.0) was used for statistical analysis. Data with a normal distribution are reported as the means ± SDs. The paired *t*-test was used to compare the differences in UBM parameters before and after LPI. The AOD750 after LPI and baseline parameters AOD750, angle opening distance at 500 μm from the scleral spur (AOD500), AOD750, angle recess area at 750 μm from the scleral spur (ARA750), ACD, LV, IT750, IT2000, IC, CBT0, TCPD750, and ICPD750 were analyzed by univariable and multiple linear regression analysis. *P* <0.05 was considered statistically significant.

## Results

### Demographics and Baseline Clinical Characteristics of the Study Population

A total of 100 subjects were analyzed, including 38 with the pupillary block type (38.0%), 12 with the plateau iris configuration (12.0%), and 50 with the mixed mechanism type (50.0%) (Table 1). The mean age across all participants was 58.14 years (range, 33–74 years; standard deviation, 8.20 years), and the majority were females (74.0%).

**Table 1:** Demographics and Baseline demographic and clinical examination.

**Varaible**	**Pupillary block (*N* = 38)**	**Plateau iris (*N* = 12)**	**Mixed mechanism (*N* = 50)**	***P*-value**
Age, yr, mean (SD)	59.82 (7.79)	57.42 (7.56)	57.04 (8.58)	>0.05
Female gender,%	81.6%	50.0%	74.0%	<0.001

### Univariable and Multivariable Linear Regression Analysis for Baseline Predictors of AOD750 After Laser Peripheral Iridotomy

Linear regression analyses were performed to determine the baseline parameters that predict post-procedure angle widening, defined as AOD750 after LPI (Table 2). Greater baseline AOD500, AOD750, TCPD750, ARA750, ACD, and LV predicted higher odds of angle widening after LPI (*P* <0.05 for all). Multiple linear regression (*R*^2^ = 0.427, *P* <0.001) showed that greater baseline IT750 (*P* <0.001) was associated with a lower likelihood of angle widening, whereas greater baseline AOD750 (*P* = 0.001), ACD (*P* = 0.001) and LV (*P* = 0.015) predicted a higher likelihood of angle widening after LPI (P <0.001).

**Table 2:** Univariable and multivariable linear regression analysis for baseline predictors of AOD750 after laser peripheral iridotomy.

	**Univariate**			**Multivariate**		
**Baseline parameters**	**B(SE)**	**β**	*P*-value^*^	**B(SE)**	**β**	*P*-value^*^
Age	−1.639 (1.955)	−0.086	0.404			
Gender	22.000 (36.429)	0.062	0.547			
AOD250	0.272 (0.176)	0.158	0.126			
AOD500	0.709 (0.161)	0.415	<0.001			
AOD750	0.570 (0.112)	0.467	<0.001	0.381 (0.110)	0.312	0.001
IT750	‘−0.698 (0.221)	−0.312	0.002	‘−0.865 (0.183)	−0.386	<0.001
IT2000	‘−0.168 (0.183)	−0.095	0.362			
TCPD750	0.285 (0.094)	0.300	0.003			
ICPD750	0.145 (0.075)	0.197	0.055			
ARA750	0.001 (0.000)	0.306	0.003			
IC	‘−110.303 (182.231)	−0.063	0.546			
ACD	213.936 (57.993)	0.357	<0.001	180.882 (54.004)	0.302	0.001
ACW	2.551 (22.410)	0.012	0.910			
LV	108.065 (41.185)	0.263	0.010	83.119 (33.662)	0.202	0.015
CBT0	92.747 (94.634)	0.100	0.330			

* *P <0.05 indicate statistical significance*.

*AOD250, angle opening distance at 250 μm from the scleral spur; AOD500, angle opening distance at 500 μm from the scleral spur; AOD750, angle opening distance at 750 μm from the scleral spur; IT750, iris thickness at 750 μm from the scleral spur; IT2000, iris thickness at 2000 μm from the scleral spur; TCPD750, trabecular-ciliary process distance at 750 μm from the scleral spur; ICPD750, iris-ciliary process distance at 750 μm from the scleral spur; ARA750, angle recess area at 750 μm from the scleral spur; IC, iris curvature; ACD, anterior chamber depth; ACW, anterior chamber width; LV, lens vault; CBT0, ciliary body thickness at the point of the scleral spur*.

The predictive equation was thus generated as follows:

$$\begin{array}{l} \text{After }-\text{ LPIAOD750 }=\text{ 177}.\text{340 }+\text{ 180}.\text{882}{\;}^{*}\text{ ACD }-\text{ 0}.\text{865}\\ \;{\;}^{*}\;IT750\;+\;0.381^{*}AOD750\\ \text{ + 83}.\text{119}{\;}^{*}\text{ LV} \end{array}$$

### Stepwise Multiple Linear Regression Analysis of AOD750 in the Different Angle Closure Groups

Table 3 presents the results of the stepwise multiple linear regression analyses for factors associated with AOD750 after LPI. The equation consisted of four parameters (ACD, IT750, AOD750, and LV) and explained 42.7% of the variability in AOD750. In the mixed mechanism group, AOD750 was the only significant variable (*P* <0.05), explaining 11.4% of the variability in AOD750. In the Pupillary block, the equation consisted of two parameters (AOD750 and ICPD750) explaining 54.2% of the variability in AOD750. In the plateau iris configuration group, the equation consisted of two parameters (IT750 and AOD250), explaining 68.6% of the variability in AOD750.

**Table 3:** Stepwise multiple linear regression analysis of AOD750 in the different angle-closure groups.

**Variables in group**	**Variable**	**Regression**	**Equation**
		**coefficient(β)**	**R squared**
Total	IT750	−0.386	0.427
	AOD750	0.312	0.427
	LV	0.202	0.427
	ACD	0.302	0.427
Pupillary block	AOD750	0.771	0.542
	ICPD750	0.347	0.542
Mixed mechanism	AOD750	0.477	0.114
plateau iris configuration	IT750	−0.639	0.686
	AOD250	0.590	0.686

*IT750, iris thickness at 750 μm from the scleral spur; AOD750, angle opening distance at 750 μm from the scleral spur; LV, lens vault; ACD, anterior chamber depth; ICPD750, iris-ciliary process distance 750; AOD250, angle opening distance at 250 μm from the scleral spur*.

### Changes in Mean Anterior Segment Parameters Before and After Laser Peripheral Iridotomy

All angle parameters increased after LPI, including mean angle opening distance at 250 μm from the scleral spur (AOD250) (160.84 μm pre-procedurally vs 232.68 μm post-procedurally; P <0.001) mean AOD500 (205.68 μm pre-procedurally vs. 311.94 μm post-procedurally; P <0.001); mean AOD750 (297.98 μm pre-procedurally vs. 460.33 μm post-procedurally; *P* <0.001); and mean ARA750 (125094.63 μm^2^ pre-procedurally vs. 176316.46 μm^2^ post-procedurally; *P* <0.001). For all angle parameters, the ACW increased after LPI (11.24 mm pre-procedurally vs. 11.46 m post-procedurally; *P* <0.001). Among iris parameters and ciliary body parameters, IT at 2,000 μm (326.45 μm pre-procedurally vs. 360.85 μm post-procedurally; P = 0.01), IC (0.28 mm pre-procedurally vs. 0.01 mm post-procedurally; *P* <0.001), and ICPD (586.32 μm pre-procedurally vs. 459.61 μm post-procedurally; *P* <0.001) were lower after LPI (Table 4).

**Table 4:** Changes in mean anterior segment parameters before and after laser peripheral iridotomy.

**Parameter**	**Pre-LPI mean (SD)**	**Post-LPI mean (SD)**	*P*-value^*^
**Angle parameters**			
AOD250 (μm)	160.84 (91.29)	232.68 (108.71)	<0.001
AOD500 (μm)	205.68 (91.81)	311.94 (120.73)	<0.001
AOD750 (μm)	297.98 (128.60)	460.33 (156.94)	<0.001
ARA750 (μm^2^)	125094.63 (54632.66)	176316.46 (62266.55)	<0.001
**Anterior chamber parameters**			
ACD (mm)	2.07 (0.26)	2.09 (0.26)	0.062
LV (mm)	0.44 (0.38)	0.47 (0.42)	0.079
ACW (mm)	11.24 (0.73)	11.46 (0.77)	0.004
**Iris parameters**			
IT750 (μm)	280.59 (70.05)	289.59 (70.30)	0.212
IT2000 (μm)	326.45 (88.45)	360.85 (99.10)	0.002
IC (mm)	0.28 (0.09)	0.01 (0.04)	<0.001
**Ciliary body parameters**			
CBT0 (mm)	0.99 (0.17)	1.00 (0.16)	0.830
TCPD750 (μm)	1031.57 (165.38)	1033.14 (153.22)	0.920
ICPD750 (μm)	586.32 (212.96)	459.61 (202.77)	<0.001

* *P <0.05 represented statistical significance*.

*AOD250, angle opening distance at 250 μm from the scleral spur; AOD500, angle opening distance at 500 μm from the scleral spur; AOD750, angle opening distance at 750 μm from the scleral spur; ARA750, angle recess area at 750 μm from the scleral spur; ACD, anterior chamber depth; LV, lens vault; ACW, anterior chamber width; IT750, iris thickness at 750 μm from the scleral spur; IT2000, iris thickness at 2,000 μm from the scleral spur; IC, iris curvature; CBT0, ciliary body thickness at point of the scleral spur; TCPD750, trabecular-ciliary process distance at 750 μm from the scleral spur; ICPD750, iris-ciliary process distance at 750 μm from the scleral spur*.

## Discussion

In a recent study, He (11) noted that patients identified through community-based screening faced no immediate threat to their vision, and so widespread prophylactic laser peripheral iridotomy was not recommended. However, we speculated that patients identified through hospital-based screening might have different results. AOD750 was found to be associated with gonioscopic angle closure; therefore, we proposed a formula to predict the variability in AOD750 after LPI.

In previous studies, only one or two baseline predictive parameters were found for AOD or the percentage change in mean AOD after laser peripheral iridotomy. For example, the equation of How (7) explained 13% of the variability of the percentage change in mean AOD750; the equation of Zebardast (9) explained 34% of the variability of the change in AOD750 before and after the operation; and Atalay (12) found that in APAC patients, baseline iris thickness explained one-third of the variability of the change in AOD750. They found that post-procedural AOD750 was related to baseline parameters such as LV, IT750, IOP, PD, male sex, axial length, angle width, ACD and IC. These equations all explained less of the change in AOD750 than the one generated in this study, which fit the data well and explained 42.7% of the variability of AOD750 after LPI.

In contrast to previous studies based on anterior-segment -OCT, our study included ciliary body parameters such as TCPD and ICPD by using UBM. Linear regression analyses determined that greater baseline TCPD750 predicted higher odds of AOD750 after LPI (*P* <0.05). Our study included four baseline parameters in the final equation, which enabled superior accuracy.

In the multivariable analysis equation, the larger the baseline ACD, LV, and AOD750 before the operation, the wider the angle was after LPI, which may be due to the larger anterior chamber depth and the narrower distance between the trabecular meshwork and the ciliary body at baseline, which allows the angle to become flatter after LPI, thus leading to greater angle opening.

In addition, among the subgroups with different angle-closure mechanisms, we found that the prediction equation for the plateau iris configuration subgroup was the most accurate, followed by the pupillary block subgroup and the mixed mechanism subgroup. LPI may have a limited ability to induce angle opening in patients with a plateau iris configuration. The AOD750 after LPI depended only on the size of AOD250 and IT750 at baseline. Recent studies have suggested that LPI alone has a limited therapeutic effect on patients with plateau iris configuration and may need to be combined with laser peripheral iridoplasty (LPIP), which is consistent with our findings (10, 13).

In this study, 100 patients with PACS were observed. It was found that the anterior chamber angle was widened, the ACD was deepened, the IC was decreased and the distance between the iris and the ciliary body was significantly shortened after LPI in a Guangzhou population with PACS, which is consistent with previous research results (14–17). The angle opening distance was related to the baseline ACD, IT750, AOD750, and LV before LPI. In the pupillary block subgroup, there was a significant correlation between the angle opening distance after LPI and the baseline IC, which is consistent with previous studies. Previous studies have suggested that greater baseline IC is associated with a higher likelihood of angle widening (8, 16).

The limitations of this study are as follows. Although AOD750 was used as the standard in this study, the magnitude of AOD750 does not directly define the quality of LPI. Second, two different senior doctors performed the gonioscopy, and, as in other studies, there must inevitably exist some inter-observer variability due to the subjective nature of gonioscopy. Third, our equation was based on univariable and multivariable linear regression equations. However, some baseline parameters may have no linear correlation with the AOD or percentage change in mean AOD after LPI. This may have caused us to miss some important baseline predictors. We will conduct further prospective studies to verify the reproducibility and accuracy of the proposed equation. Finally, non-standardized UBM gain parameters may affect the accuracy, reproducibility and further analysis of the results.

In conclusion, our study found that AOD750 was correlated with baseline ACD, IT750, AOD750 and LV, and we could predict the post-procedural AOD angle according to the structural parameters of the pre-procedural angle. Our findings could serve as a valuable reference to inform clinical work.

## Data Availability Statement

The original contributions presented in the study are included in the article/supplementary material, further inquiries can be directed to the corresponding author/s.

## Ethics Statement

The studies involving human participants were reviewed and approved by Ethical Committee of Zhongshan Ophthalmic Center. Written informed consent for participation was not required for this study in accordance with the national legislation and the institutional requirements.

## Author Contributions

ML and XG: design and conduct of the study. LC and HH: collection of data. YZ and CZ: analysis of data. JR, HL, YL, and HG: preparation of the manuscript. All authors review and final approval of the manuscript.

## Conflict of Interest

The authors declare that the research was conducted in the absence of any commercial or financial relationships that could be construed as a potential conflict of interest.

## Publisher's Note

All claims expressed in this article are solely those of the authors and do not necessarily represent those of their affiliated organizations, or those of the publisher, the editors and the reviewers. Any product that may be evaluated in this article, or claim that may be made by its manufacturer, is not guaranteed or endorsed by the publisher.
